# Prevalence of Mutated Colistin-Resistant *Klebsiella pneumoniae*: A Systematic Review and Meta-Analysis

**DOI:** 10.3390/tropicalmed7120414

**Published:** 2022-12-02

**Authors:** Nik Yusnoraini Yusof, Nur Iffah Izzati Norazzman, Siti Nur’ain Warddah Ab Hakim, Mawaddah Mohd Azlan, Amy Amilda Anthony, Fatin Hamimi Mustafa, Naveed Ahmed, Ali A. Rabaan, Souad A. Almuthree, Abdulsalam Alawfi, Amer Alshengeti, Sara Alwarthan, Mohammed Garout, Eman Alawad, Chan Yean Yean

**Affiliations:** 1 Institute for Research in Molecular Medicine (INFORMM), Health Campus, Universiti Sains Malaysia, Kubang Kerian 16150, Kelantan, Malaysia; 2 Faculty Science and Marine Environment, Universiti Malaysia Terengganu, Kuala Nerus 21030, Terengganu, Malaysia; 3 Department of Medical Microbiology and Parasitology, School of Medical Sciences, Universiti Sains Malaysia, Kubang Kerian 16150, Kelantan, Malaysia; 4 Molecular Diagnostic Laboratory, Johns Hopkins Aramco Healthcare, Dhahran 31311, Saudi Arabia; 5 College of Medicine, Alfaisal University, Riyadh 11533, Saudi Arabia; 6 Department of Public Health and Nutrition, The University of Haripur, Haripur 22610, Pakistan; 7 Department of Infectious Disease, King Abdullah Medical City, Makkah 43442, Saudi Arabia; 8 Department of Pediatrics, College of Medicine, Taibah University, Al-Madinah 41491, Saudi Arabia; 9 Department of Infection Prevention and Control, Prince Mohammad Bin Abdulaziz Hospital, National Guard Health Affairs, Al-Madinah 41491, Saudi Arabia; 10 Department of Internal Medicine, College of Medicine, Imam Abdulrahman Bin Faisal University, Dammam 34212, Saudi Arabia; 11 Department of Community Medicine and Health Care for Pilgrims, Faculty of Medicine, Umm Al-Qura University, Makkah 21955, Saudi Arabia; 12 Adult Infectious Diseases Department, Prince Mohammed Bin Abdulaziz Hospital, Riyadh 11474, Saudi Arabia

**Keywords:** *Klebsiella pneumoniae*, colistin resistance, mutation, systematic review, meta-analysis

## Abstract

The emergence of genetic mutations in chromosomal genes and the transmissible plasmid-mediated colistin resistance gene may have helped in the spread of colistin resistance among various *Klebsiella pneumoniae* (*K. pneumoniae*) isolates and other different bacteria. In this study, the prevalence of mutated colistin-resistant *K. pneumoniae* isolates was studied globally using a systematic review and meta-analysis approach. A systematic search was conducted in databases including PubMed, ScienceDirect, Scopus and Google Scholar. The pooled prevalence of mutated colistin resistance in *K. pneumoniae* isolates was analyzed using Comprehensive Meta-Analysis Software (CMA). A total of 50 articles were included in this study. The pooled prevalence of mutated colistin resistance in *K. pneumoniae* was estimated at 75.4% (95% CI = 67.2–82.1) at high heterogeneity (*I*^2^ = 81.742%, *p*-value < 0.001). Meanwhile, the results of the subgroup analysis demonstrated the highest prevalence in Saudi Arabia with 97.9% (95% CI = 74.1–99.9%) and Egypt, with 4.5% (95% CI = 0.6–26.1%), had the lowest. The majority of mutations could be observed in the *mgrB* gene (88%), *pmrB* gene (54%) and *phoQ* gene (44%). The current study showed a high prevalence of the mutation of colistin resistance genes in *K. pneumoniae*. Therefore, it is recommended that regular monitoring be performed to control the spread of colistin resistance.

## 1. Introduction

*Paenibacillus polymyxa*, previously known as *Bacillus polymyxa var. colistinus*, is the source of the polymyxins, polycationic peptide antibiotic class, which were first discovered in 1947 [1]. Clinically, polymyxin B and polymyxin E (also known as colistin) are two available forms of polymyxin agents [2]. Since 1959, colistin has been prescribed to people infected with Gram-negative bacteria, including *Pseudomonas aeruginosa*, *Acinetobacter baumannii*, *Klebsiella* spp., *Escherichia coli* and other *Enterobacterales* [1]. Considering colistin’s adverse side effects, including nephrotoxicity and neurotoxicity, the use of colistin rapidly decreased from the early 1970s to the early 2000s [3].

Colistin exerts its antibacterial action against Gram-negative bacteria via interaction with the lipid A, a component of the lipopolysaccharide (LPS) of the outer membrane (OM) [4]. The uniqueness of colistin’s chemical structure makes it an excellent amphipathic agent that can act in a detergent-like manner to alter the structure of the OM [3]. It attaches to the lipid A, replacing the phosphate groups, the membrane stabilizers of LPS, with divalent cations, Ca^2+^ and Mg^2+^ [1,5]. Subsequently, the bacterial membrane destabilizes, causing leakage of the cellular contents, resulting in bacterial lysis and death [3]. Recently, colistin has been reevaluated as a viable therapeutic choice for critically ill patients due to the widespread of multidrug-resistant (MDR) Gram-negative bacteria and the dearth of novel antibacterial agents [1,2]. Additionally, its effectiveness against almost all MDR Gram-negative bacteria, including *K. pneumoniae*, makes colistin a last resort drug of choice [3,6].

*K. pneumoniae* is a Gram-negative bacterium with a rod shape and is categorized under the *Enterobacteriaceae* family [7]. It causes one-third of Gram-negative infections in nosocomial and community-acquired infections globally [2,8]. A wide range of illnesses, such as bloodstream infections, wound infections, urinary tract infections (UTIs) [9], pneumonia, as well as infections at the surgical site have been reported in people infected with *K. pneumoniae*. Nowadays, it is progressively difficult to cure diseases caused by *K. pneumoniae* due to the rising incidence of antibiotic resistance isolates [5].

In 1983, *K. pneumoniae* was first reported to be resistant to beta-lactam antibiotics due to its ability to produce extended-spectrum beta-lactamases (ESBL). Hence, carbapenems were given to treat infections of this bacteria [2]. Unfortunately, carbapenem-resistant *K. pneumoniae* (CRKP) started to emerge due to the rapid spread of oxacillinase-48 (OXA-48), New Delhi metallo-beta-lactamase (NDM), and *K. pneumoniae* carbapenemase (KPC) [5]. The constant increase in the prevalence of CRKP infections as well as the limitation of treatment options has posed a serious menace to human health, therefore increasing the human mortality rates. Hence, tigecycline and colistin are used as the ultimate drug options in the treatment of MDR *K. pneumoniae* infections that are resistant to extended-spectrum cephalosporins, carbapenems, amino-glycosides, and fluoroquinolones [2,8].

Colistin-resistant *K. pneumoniae* (ColRkp) has sadly started to spread worldwide as a result of the overuse and improper use of colistin in human and animal medicine [6]. The development of colistin resistance in *K. pneumoniae* could happen due to several reasons. The most frequent mechanism is the alteration of the molecular structure of LPS, which lowers its affinity for colistin [3,10]. The genetic mutations on chromosomal genes lead to LPS modifications by inactivating the *mgrB* gene, upregulating the PhoP/PhoQ signaling system and PmrA-regulated *pmrHFIJKLM* operon [2,3]. In addition, *K. pneumoniae* is able to become resistant to colistin upon acquiring the plasmid gene, which is the *mcr* gene [3]. To date, ten mcr homologues (*mcr-1* to *mcr-10*) have been identified [11].

The discovery of mutational chromosomal genes and plasmid-mediated colistin resistance is worrying due to its potential to expedite the transmission of colistin resistance between various *K. pneumoniae* strains and different bacteria [3]. Therefore, it is critical to have a better understanding of the occurrence of mutations in ColRkp to assist in the development of more effective interventional measures that are capable of reducing the spread of MDR *K. pneumoniae*. The current systematic review and meta-analysis aim to gather the information that is currently known on colistin resistance gene mutations in *K. pneumoniae* and to estimate the global prevalence of ColRkp.

## 2. Materials and Methods

### 2.1. Search Strategy

A thorough systematic literature search was performed using the keywords: (colistin resistance gene) OR (Polymyxin-E) OR (mutation in colistin resistance gene) AND (*Klebsiella pneumoniae*) on four databases: PubMed, ScienceDirect, Scopus, and Google Scholar. Criteria such as time of publication, study design, and language were omitted from the search filters to ensure comprehensive data collection.

### 2.2. Inclusion and Exclusion Criteria

The following studies were included in our study based on the following criteria: (1) study about *K. pneumoniae*, (2) study reporting on colistin resistance gene in *K. pneumoniae*, (3) study on mutation in colistin resistance gene in *K. pneumoniae*, (4) studies written in English. Studies with insufficient information, review papers, books, case reports, media reports, short letters, and studies not reporting *K. pneumoniae* and colistin resistance genes in *K. pneumoniae* were excluded.

### 2.3. Quality Assessment

The Joanna Briggs Institute’s (JBI) critical appraisal technique for studies reporting prevalence data was used to evaluate the eligibility of the studies. The appraisal checklist consists of nine key questions, which focus on the proper sample frame, study topic, and adequate data analysis. Each response is graded as “yes”, “no”, or “unclear”. The response “yes” received a score of 1, while the responses “no” and “unclear” received scores of 0. Studies deemed to be of high quality and included in the study had scores of 7 or higher from the checklist.

### 2.4. Data Extraction

Data from relevant studies were retrieved under the following requirements: (1) author, (2) year of publication, (3) period of study, (4) country of study, (5) type of sample (human/animal/environment), (6) number of colistin resistance isolates, (7) number of mutated cases, (8) mutation detection method, (9) genes encoded for colistin resistance, (10) mutated colistin resistance genes. Studies that analyzed colistin gene mutations from more than one country were categorized as multiple countries rather than individual countries to prevent confusion during data extraction and analysis. 

### 2.5. Data Analysis

The Comprehensive Meta-Analysis Software (CMA) Version 3.0 (Biostat, Inc., Englewood, NJ, USA) was used to analyze the data on the prevalence of mutated ColRkp and colistin resistance gene mutations in ColRkp isolates. The subgroup analysis was carried out according to the country of study. A random-effects model using the DerSimonian–Laird method of meta-analysis at a 95% confidence interval (CI) was used to measure the pooled prevalence of the mutational colistin resistance gene in *K. pneumoniae*. Heterogeneity was determined using *I*^2^ test statistics. The value of *I*^2^ ≤ 25% denoted low heterogeneity, 25% < *I*^2^ ≤ 75% denoted moderate heterogeneity, and *I*^2^ > 75% denoted high heterogeneity [10]. Funnel plot diagrams and Egger’s regression test were employed to evaluate whether publication bias existed. For all tests, a *p*-value of < 0.05 was considered statistically significant.

## 3. Results

### 3.1. Search and Screening Results

**Figure 1:**
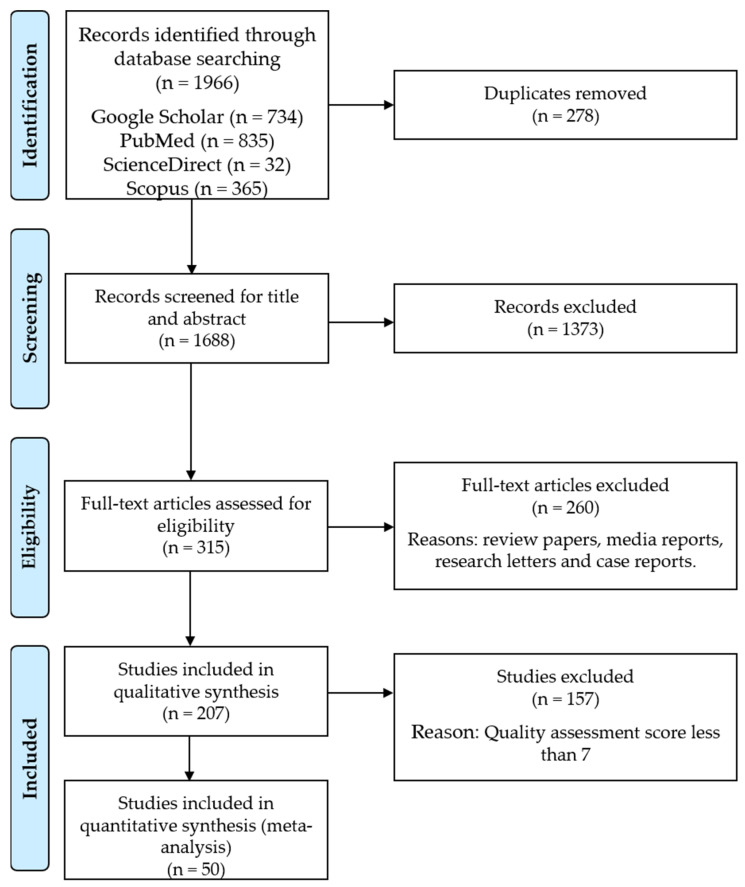
PRISMA flowchart illustrating the process of identifying, screening and selecting the eligible articles in this study.

A total of 1966 articles, as shown in Figure 1, were identified through online databases (Google Scholar = 734; PubMed = 835; ScienceDirect = 32; Scopus = 365) based on the keywords used. The screening process continued with 1688 articles involved. According to the inclusion and exclusion criteria, 1373 articles were removed from consideration after the title and abstract screening procedure, while 315 articles were accepted. Then, the remaining articles proceeded to the full-text screening process, which resulted in 207 articles being selected to proceed further for data extraction. Meanwhile, 157 articles were removed due to the high and moderate risk bias based on the quality evaluation score that was less or equal to six (≤6 score) (Supplementary Table S1). Finally, only 50 studies that portrayed all the selected criteria would be accepted for further analysis.

### 3.2. Characteristics of the Eligible Studies

**Figure 2:**
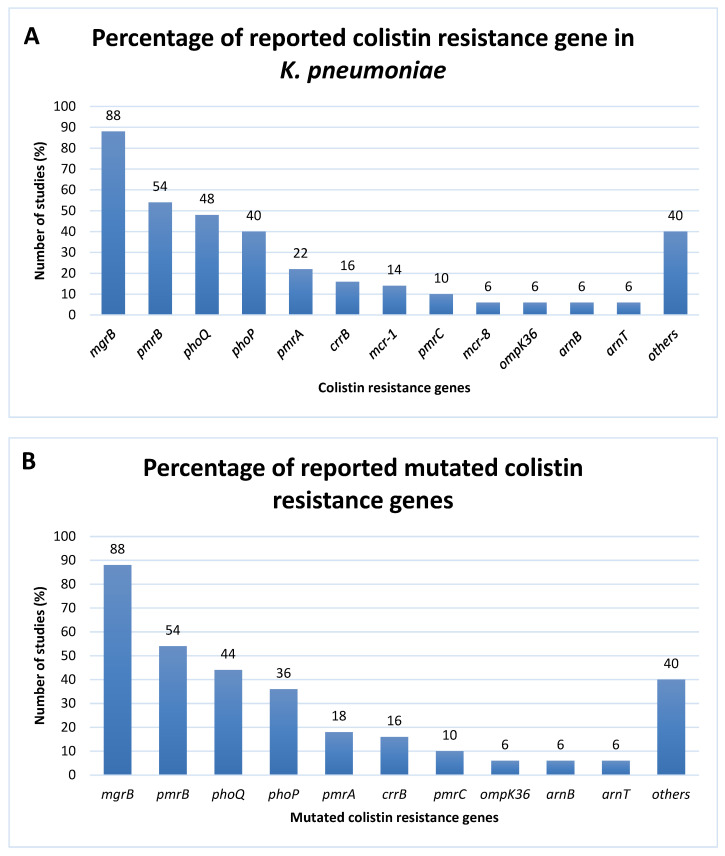
The percentage of reported (**A**) colistin resistance genes and (**B**) mutated colistin resistance genes. Others: *acrS*, *arnA*, *arnC*, *crrA*, *ompK35*, *ompK37*, *pagP*, *phoR*, *pmrE*, *pmrF*, *pmrJ*, *pmrK* and *ramR*.

All the eligible studies included in the meta-analyses were of high methodological quality (Supplementary Table S1). From 50 studies included in this review, the highest numbers were from Italy (n = 6), India (n = 5), China (n = 5) and Korea (n = 5). The genomic data of 1215 colistin-resistant isolates were analyzed for the presence of colistin resistance genes and mutated colistin resistance genes (Table 1). Of 1215, there were 1203 and 12 isolated from humans and animals, respectively. The most frequently reported colistin resistance genes were *mgrB* gene (n = 44, 88%), *pmrB* gene (n = 27, 54%), *phoQ* gene (n = 24, 48%), *phoP* gene (n = 20, 40%) and *pmrA* gene (n = 11, 22%) (Figure 2A). The *K. pneumoniae* isolates have been found to acquire mobilized colistin resistance (*mcr*) genes. Two types of *mcr* genes were also reported, *mcr-1* (n = 7, 14%) and *mcr-8* (n = 3, 6%) genes. Out of 1215 isolates, 836 isolates (824 from humans and 12 from animals) were found to have mutations in their genes associated with colistin resistance. The most common method for determining mutations in colistin resistance genes of *K. pneumoniae* isolated from all the studies included in this systematic review and meta-analysis study was DNA sequencing (Sanger, whole-genome or next generation sequencing). From the mutational data analyzed (Figure 2B), the studies detected mutations in the *mgrB* gene (88%), *pmrB* gene (54%), and *phoQ* gene (44%). The *ompK36* gene, *arnB* gene and *arnT* gene had the same percentage which was 6%. The detected mutations are listed in Supplementary Table S2.

### 3.3. The Pooled Prevalence of Mutated Colistin-Resistant K. pneumoniae (ColRkp)

**Figure 3:**
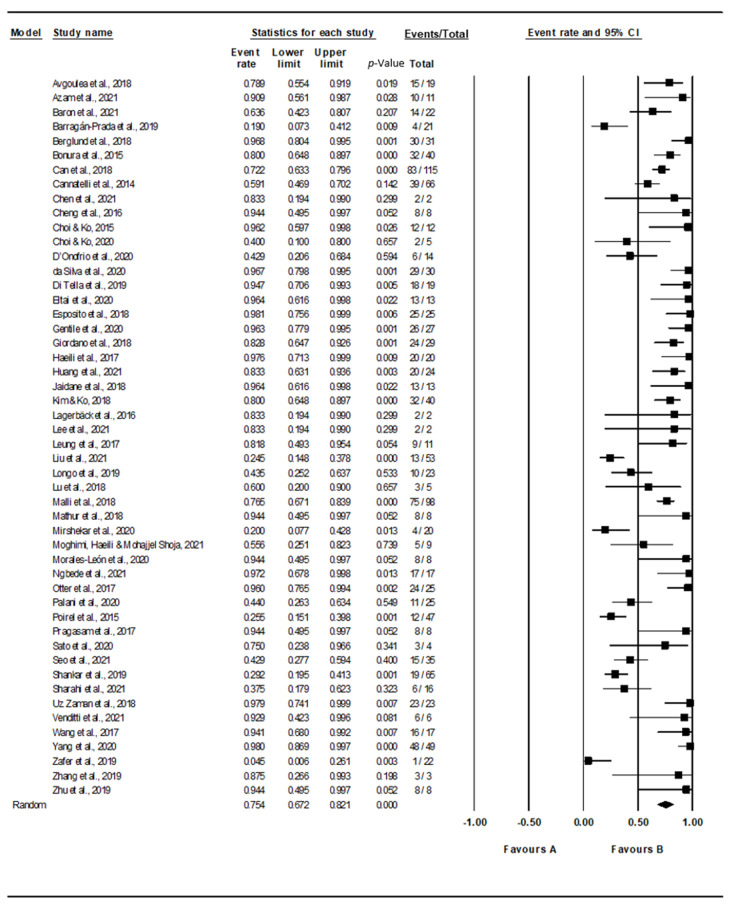
Forest plot showing the pooled prevalence of mutated colistin-resistant *K. pneumoniae* (ColRkp) estimated by a random effect model of meta-analysis (75.4%, *I*^2^ = 81.742, 95% CI = 67.2–82.1, *p*-value < 0.001). The event rate was calculated to report the summary effect size. Studies are displayed as squares, and size of the square indicates the weight given to the study in meta-analysis using CMA software [12,13,14,15,16,17,18,19,20,21,22,23,24,25,26,27,28,29,30,31,32,33,34,35,36,37,38,39,40,41,42,43,44,45,46,47,48,49,50,51,52,53,54,55,56,57,58,59,60,61].

**Figure 4:**
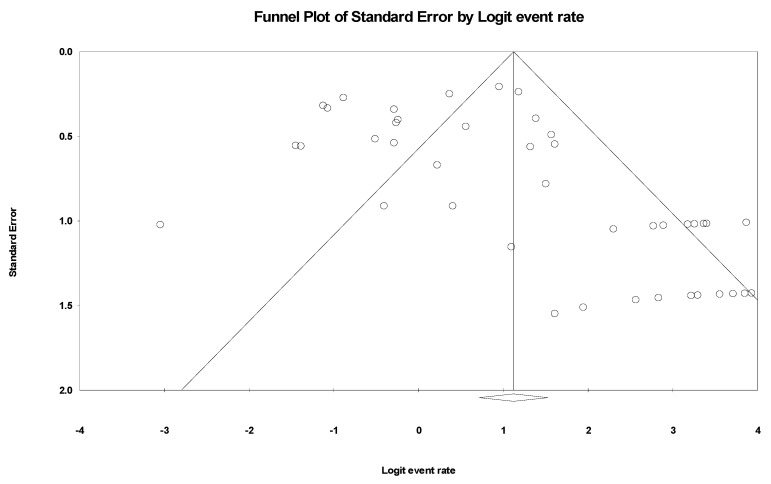
Funnel plot showing the evidence of publication bias.

Based on the random-effect model, the pooled prevalence of ColRkp mutations was estimated at 75.4% (95% CI = 67.2–82.1), with high heterogeneity (*I*^2^ = 81.742%, *p*-value < 0.001) (Figure 3). However, publication bias was observed, as represented in the asymmetrical funnel plot (Figure 4). Therefore, Egger’s test was used to evaluate the extent of bias. The result of this test revealed a significant publication bias (*p*-value < 0.001). 

### 3.4. Subgroup Meta-Analysis

The subgroup analysis was carried out according to the country of the included studies. Fifteen countries with low heterogeneity were Chile, Croatia, Egypt, France, Greece, Japan, Nigeria, Qatar, Saudi Arabia, Spain, Tunisia, Turkey, United Kingdom, United States, and Vietnam (*I*^2^ = 0%). The result of the analysis (Table 2) showed that Saudi Arabia (n = 1) with 97.9% (95% CI = 74.1–99.9%) had the highest pooled prevalence and Egypt (n = 1) with 4.5% (95% CI = 0.6–26.1%) had the lowest. Interestingly, India and Korea, possessing the same number of studies (n = 5), had a similar prevalence, which was 68.6% (95% CI = 37.3–88.9%). Meanwhile, the heterogeneity was highest among studies conducted in multiple countries (n = 2) (*I*^2^ = 91.559%, *p*-value = 0.001) followed by Brazil (n = 2) (*I*^2^ = 90.805%, *p*-value = 0.001), India (n = 5) (*I*^2^ = 79.819%, *p*-value = 0.001), China (n = 5) (*I*^2^ = 79.333%, *p*-value = 0.001) and Iran (*I*^2^ = 75.566%, *p*-value = 0.006). 

## 4. Discussion

Specific mutations in the chromosomal genes and transmissible plasmid genes are associated with colistin resistance in *Enterobacteriaceae*, including *K. pneumoniae* [62]. To the best of our knowledge, this is the first report evaluating the prevalence of mutation in colistin resistance genes in ColRkp worldwide. 

In this study, 1215 ColRkp isolated from 23 countries between 2006 and 2020 were studied. The majority of ColRkp was isolated from humans (99%) and the rest was collected from animals. All isolates from animals were found to have mutations in the colistin resistance genes, whereas 68.50% of human isolated-ColRkp carried mutated colistin resistance genes. Based on our meta-analysis, the pooled prevalence of mutated ColRkp isolated from humans and animals was 75.4%. The high pooled prevalence may be the result of over-prescription of colistin in human medicine, global trade and travel to endemic countries [63]. Similarly, the long-term use of colistin in veterinary medicine may have contributed to the rise in the number of colistin-resistant isolates. 

Based on country, the highest pooled prevalence was recorded in Saudi Arabia with 97.9% (95% CI = 74.1–99.9%). This result was supported by a study conducted in King Fahad Hospital, Medina, which also showed high resistance rates for colistin, 40.7% [64]. Colistin was known to be used as an alternative treatment to treat *K. pneumoniae* infection in Saudi Arabia when the first-choice treatments, carbapenems, imipenem and meropenem, were not effective for treating the infected patients. However, this value should be interpreted cautiously since only one study was reported in this country (n = 1). The low number of the studies included in the meta-analysis might cause over-estimation or low-estimation of pooled prevalence. Similarly, Vietnam (96.8%, 95% CI = 80.4–99.5%), Tunisia (96.4%, 95% CI = 61.6–99.8%), Qatar (96.4%, 95% CI = 61.6–99.8%), United Kingdom (96.0%, 95% CI = 76.5–99.4%) and Chile (94.4%, 95% CI = 49.5–99.7%) also reported the high pooled prevalence, but the number of studies conducted in these countries were also limited (n = 1). 

It was found in the current study that eight countries in the Asian region showed high prevalence (>50%) of mutated colistin-resistant *K. pneumoniae*. This was followed by four countries in the Middle East (Saudi Arabia, Qatar, Tunisia and Iran) and Europe (France, Greece, Italy and United Kingdom), two in South America (Brazil and Chile), and one in Africa (Nigeria). Meanwhile, two European (Spain and Croatia) countries and one Middle Eastern (Egypt) country showed low prevalence (< 50%) of these strains. These data demonstrate the global dissemination and evolution of colistin resistance in *K. pneumoniae*, highlighting the necessity to evaluate antimicrobial resistance (AMR) management strategies internationally rather than localized ones. Controlling AMR necessitates looking at more than simply the quantity of antibiotics used, the types of antibiotics utilized, and the patterns of antibiotic use. There is an immediate need to learn more about the spread of AMR and how the current social, economic, and policy settings facilitate its development. 

Specifically, the majority of the data was from Asia, with the highest number of reports (21/50; 42%). This was followed by Europe (11/50; 22%), the Middle East (7/50; 14%), America (5/50; 10%) and Africa (1/50; 2%). Most studies were conducted in urban areas, which have a greater accessibility to antimicrobial drugs and the mutational pattern of colistin resistance in rural areas may differ. Additionally, policies of conducting antimicrobial susceptible testing will differ regionally across hospitals and, frequently, these policies will not be applied in a consistent manner.

According to the analyses, there was significant high heterogeneity, *I*^2^ = 85.785%. Hence, subgroup analysis based on the country of the study was performed to discover the sources of heterogeneity. As a result, we were able to reduce the effect of heterogeneity for some countries, except for multiple countries (*I*^2^ = 91.559%, *p*-value = 0.001) and Brazil (*I*^2^ = 90.805%, *p*-value = 0.001) that reported higher heterogeneity compared to the pooled prevalence’s heterogeneity. We postulated that the diverse mutation detection methods used in each study may have contributed to the high heterogeneity. Some studies used both molecular techniques (including PCR, WGS and Sanger sequencing) and bioinformatics analysis (such as ResFinder, and ISfinder) while other studies used either molecular methods or bioinformatics tools to detect mutations in colistin resistance genes. Therefore, it is critically important to ensure which method provides high sensitivity and specificity for detecting both all currently known- and new-mutation colistin resistance genes in the future to improve our understanding of colistin resistance mechanisms. 

Most of the reported mutations occurred in the *mgrB* gene (88%), followed by the *pmrB* gene (54%), *phoQ* gene (44%), *phoP* gene (36%) and *pmrA* gene (18%) (Figure 2B). The significant number of research reports on these genes’ alterations could be attributed to their high relationship with colistin-resistant isolates. Both *pmrA* and *pmrB* genes (encoded for PmrA and PmrB, respectively) work together via the PmrAB two-component system (TCS). The activated PmrAB TCS activates the transcription of the *pmr*CAB and *pmr*HFIJKLM operons, which subsequently cause LPS modifications with the addition of 4-amino-4-deoxy-L-arabinose (L-Ara4N) or phosphoethanolamine (pEtN) [7,63]. Another TCS is PhoPQ encoded by *phoP* and *phoQ* genes. The activated PhoPQ TCS activates the transcription of *pmr*HFIJKLM operon, resulting in modification of LPS with L-Ara4N. At the same time, the phosphorylated PhoP (the activated form of PhoP protein) also promotes the activation of PmrA via PmrD, a connector protein, hence, upregulating the PmrAB signal indirectly. Interestingly, the PhoPQ is regulated by MgrB protein (also called YobG), a small regulatory transmembrane protein constituted of 47 amino acids encoded by the *mgrB* gene [6,7]. MgrB acts as a negative feedback regulator of the PhoPQ TCS [6]. It represses PhoQ thus, the phosphorylation of PhoP represses and reduces the production of pEtN [6,7].

The genetic alterations of the *mgrB* gene are well-characterized to be responsible for acquiring colistin resistance in *K. pneumoniae* [7,65,66,67]. Furthermore, judging from the significant number of reports of mutations in the *mgrB* gene (88%), this mechanism seems to be the most common colistin resistance. Mutations in the *mgrB* gene upregulate the PhoPQ TCS and consequently activate the *pmr*HFIJKLM operon, promoting overproduction of L-Ara4N that will block the binding of colistin to LPS [63]. Various non-sense mutations causing premature termination, frameshift deletions, partial or complete deletions of *mgrB* locus, stop codon mutations leading to truncated gene products and amino acid substitutions were reported in the eligible study (refer to Supplementary Table S2) causing resistance to colistin. 

It has been reported that insertional inactivation of the *mgrB* gene can be responsible for the acquired colistin resistance in *K. pneumoniae* [6]. Inactivation of the *mgrB* gene by insertion sequences (IS) of ISKpn25, ISKpn26, IS903, and ISCs68, IS5, or ISKpn14 has already been reported to cause colistin resistance in *K. pneumoniae* strains [6,68]. Our data showed insertion elements IS1, ISKpn14, IS903, IS903B, ISKpn28, IS10R, ISKpn26, IS5, IS26, IS1R, IS102, ISEc68, ISEcp1, IS1F, IS3, ISKpn25, IS10, ISKpn18, ISKpn13 were reported, with IS5 being the most commonly reported (Supplementary Table S2). IS are small (~0.7 to ~2.5 kbp), mobile genetic elements found in most bacterial genomes including *K. pneumoniae* whose presence can bring severe threat to the genome structure and gene expression [69]. IS5 element insertions in colistin-resistant derivatives are most likely endogenous, as they already exist in susceptible parental strains’ genomes [69]. Based on this finding, it reveals that the insertion of an IS5 may modulate the expression and/or function of *mgrB*, hence promoting colistin resistance in *K. pneumoniae* following exposure to colistin [23]. In this review, the insertion of various IS elements in the *mgrB* gene was observed to cause inactivation or truncation of *mgrB*, leading to loss-of-function of MgrB. IS elements are thought to be essential to adaptive evolution in bacteria by promoting genetic diversity [58]. Thus, it is crucial to keep updated on the mutations in the *mgrB* gene by IS monitoring in *K. pneumoniae* which might halt the spread of colistin resistance and reduce the risk for treatment failure [58]. 

Mutations in *pmrB* (54%) were more commonly reported than in *pmrA* (18%). According to Huang et al. [70], there are at least 70 nonsynonymous substitutions in *pmrB* related to the acquisition of colistin resistance. The substitution of threonine to proline at position 157 (Thr157Pro) in *pmrB* was highly reported in the eligible studies (Supplementary Table S2). A study comparing colistin-susceptible with colistin-resistant isolates collected from the same patient identified proline in the resistant isolate at position 157 instead of threonine (found in the susceptible isolate and other wild-type strains) [71]. Other studies also discovered the same mutations in their colistin-resistant isolates [72,73,74], thus strengthening the hypothesis that Thr157Pro plays an important role in acquiring colistin resistance. In *pmrA*, amino acid substitution Gly53Cys has been reported to confer resistance to colistin in *K. pneumoniae* [7,70]. However, in this review, only one study reported a mutation of Gly53Cys in ColRkp isolates [32]. 

Furthermore, Thr151Ala, Leu26Gln, and Arg114Ala mutations in the *phoP* gene were the most frequently reported in the included studies (Supplementary Table S2). Both Leu26Gln (in the N-terminal receiver domain of PhoP) and Arg114Ala mutations are known to confer colistin resistance in *K. pneumoniae* [70,74,75]. In contrast, there is no evidence of Thr151Ala mutation causing colistin resistance, even though it was detected in ColRkp isolates. On the other hand, in phoQ, the Asp150Gly mutation was widely observed in the eligible studies (Supplementary Table S2) and was known to cause colistin resistance [76,77]. Interestingly, this mutation was also found in the PhoQ periplasmic domain (PD) of *Salmonella enterica*, serotype *typhimurium*, resulting in higher levels of PhoQ [78].

Moreover, CrrA/CrrB is another TCS whose function appears to be linked to the PmrA/PmrB regulatory system. The function of the CrrA/CrrB TCS is unknown, but the CrrA/CrrB TCS likely influences the PmrA/PmrB TCS through CrrC, a connector protein. Some *K. pneumoniae* strains have CrrA/CrrB, and it has been noted those mutations in the *crrB* gene cause higher MICs of colistin [7]. According to our extracted data, both the *crrA* gene (4%) and the *crrB* gene (16%) (Figure 2) were found in ColRkp recorded in the included studies in which both of them were mutated. Mutations in the *crrB* gene that lead to amino acid substitution were reported in the eligible studies, including Leu94Met, Gln10Leu, Tyr31His, Trp140Arg, Asn141Ile, Pro151Ser, Ser195Asn and others (Supplementary Table S2). 

Other than that, the plasmid-mediated colistin-resistant *mcr* gene is known to be one of the mechanisms of acquired colistin resistance [79]. The plasmid-mediated *mcr* gene was responsible for the horizontal transfer of colistin resistance, and was described recently in *Enterobacteriaceae* worldwide [9]. The *mcr-1* gene was first discovered in 2015 in *K. pneumoniae* and *E. coli* isolates from China [7]. The *mcr-1* gene encodes pEtN transferase, which adds pEtN to the lipid A moiety [7,64]. In this study, aside from the *mcr-1* gene (14%), the *mcr-8* gene (6%) was also reported in the included studies (Figure 2A).

## 5. Study Limitations

Even though we have systematized the data on the occurrence of the mutation of ColRkp, this study has a few limitations. First, due to a lack of resources in some countries, this study was unable to cover all countries in order to present a thorough overview of the prevalence of the ColRkp mutation. Second, the number of studies from some countries was exceptionally high or limited, which may affect the total estimate. In addition, the majority of the isolates were from human samples, but there were very few isolates from animal samples and none of environmental samples that were eligible for the inclusion criteria; hence, a subgroup analysis incorporating the source of samples to assess and compare the prevalence between the source of samples could not be performed as it may reduce the power of the analysis. The case reports and short communications were not included in the current study, which may have led to some data being overlooked. Furthermore, we searched data from a limited number of databases for our systematic review and meta-analysis. Articles that have appeared in other databases or that are not indexed in the indices searched may have been ignored. We have also only included items published in English; as a result, publications in other languages may have been overlooked. 

## 6. Conclusions

In this study, a systematic review and meta-analysis study were conducted to report the worldwide prevalence of the mutation in the colistin resistance gene, estimated at 75.4%, which is considerably high. The estimated point is nevertheless a good indicator of the prevalence of the mutation in the colistin resistance gene globally, despite the considerable heterogeneity (*I*^2^ = 85.785%) that was found. Chromosome-related gene mutations, such as those in the *mgrB*, *pmrB*, *phoQ*, *phoP* and *pmrA* genes, were frequently observed. Additionally, the acquisition of the *mcr-1* and *mcr-8* genes was documented in the studies that qualified. It was thought that the colistin resistance in *K. pneumoniae* was caused by the mutation of these genes and the acquisition of genes from plasmids. The prevalence of this mutation must therefore be periodically evaluated in order to stop the development of ColRkp from spreading further, since its potential impact and a lack of treatment options may result in future danger. Furthermore, ColRkp belonging to sequence types often associated with human diseases (ST11, ST37 and ST15) were found among the commensal bacteria of food animals [25]. This poses a serious danger due to the possibility of these pathogens being transmitted to humans via the food chain or direct exposure.

In light of the fact that effective antimicrobial therapy has not yet been determined for ColRkp, it is important to carefully review the antimicrobial chosen for treatment. With no new antimicrobials on the horizon for treating resistant healthcare-associated infections, it is also imperative that effective preventative programs and adequate personnel be implemented to stem the tide of ColRkp. In order to control and prevent the resilience of drug-resistant pathogens, the development of rapid, low-cost, and accurate detection of determinants and mutations conferring resistance is urgently needed for routine applications of molecular analyses and to improve patient management by identifying the optimum treatment options. Consequently, it is believed that unnecessary procedures and the improper use of antibiotics should be avoided in the healthcare setting for better infectious disease surveillance and management. The high antimicrobial resistance rates are among the most serious health threats worldwide. The current study provides a thorough description of prevalence of colistin resistance among *K. pneumoniae* isolates throughout the world with the addition of the prevalence of drug resistance genes. Hence, it will help to the authorities to take some necessary measures in order to control antimicrobial resistance.

## Figures and Tables

**Table 1:** Characteristics of the 50 included studies in this review.

No.	Study ID (ref)	Country of Study	Period of Study	Source of Sample	No. of Colistin-Resistant *K. pneumoniae*	No. of Mutated Cases	Mutation Detection Method	Genes Encoded for Colistin Resistance	Mutated Colistin Resistance Genes
1	Avgoulea et al., 2018 [12]	Greece	2012–2014	Human	19	15	WGS, ResFinder	*mgrB* (15)	*mgrB* (15)
2	Azam et al., 2021 [13]	India	2017–2018	Human	11	10	PROVEAN, PCR	*mgrB* (4), *phoP* (1), *phoQ* (4), *pmrA* (1), *pmrB* (7)	*mgrB* (4), *phoP* (1), *phoQ* (4), *pmrA* (1), *pmrB* (7)
3	Baron et al., 2021 [14]	France	2014–2017	Human	22	14	WGS, PROVEAN	*acrS* (12), *crrB* (10), *mgrB* (2), *phoP* (1), *phoQ* (2), *pmrA* (13), *pmrB* (11)	*acrS* (12), *crrB* (10), *mgrB* (2), *phoP* (1), *phoQ* (2), *pmrA* (13), *pmrB* (11)
4	Barragán-Prada et al., 2019 [15]	Spain	2014–2015	Human	21	4	WGS, PCR, Sanger sequencing, ISMapper	*mgrB* (3), *pmrA* (1), *pmrB* (1)	*mgrB* (3), *pmrA* (1), *pmrB* (1)
5	Berglund et al., 2018 [16]	Vietnam	2015	Human	31	30	WGS, ResFinder, Sanger sequencing, ISFinder	*mgrB* (31)	*mgrB* (30)
6	Bonura et al., 2015 [17]	Italy	2014	Human	40	32	PCR, sequencing	*mgrB* (40)	*mgrB* (32)
7	Can et al., 2018 [18]	Turkey	2015–2016	Human	115	83	Sequencing	*mgrB* (83)	*mgrB* (83)
8	Cannatelli et al., 2014 [19]	Multiple countries	2010–2012	Human	66	39	PCR	*mgrB* (66)	*mgrB* (39)
9	Chen et al., 2021 [20]	China	2020	Human	2	2	WGS, ResFinder, ISfinder	*mgrB* (2)	*mgrB* (2)
10	Cheng et al., 2016 [21]	Taiwan	NA	Human	8	8	PCR, sequencing	*crrB* (8)	*crrB* (8)
11	Choi & Ko, 2015 [22]	Korea	NA	Human	12	12	Sequencing	*phoP* (4), *phoQ* (12), *pmrB* (12)	*phoP* (4), *phoQ* (12), *pmrB* (12)
12	Choi & Ko, 2020 [23]	Korea	2006–2007	Human	5	2	WGS	*mgrB* (2), *phoQ* (1)	*mgrB* (2), *phoQ* (1)
13	da Silva et al., 2020 [24]	Brazil	2015–2016	Human	30	29	WGS, ISfinder	*mgrB* (29)	*mgrB* (29)
14	Di Tella et al., 2019 [25]	Italy	2014–2017	Human	19	18	PCR, Sanger sequencing	*mgrB* (18)	*mgrB* (18)
15	D’Onofrio et al., 2020 [26]	Croatia	2013–2018	Human	14	6	WGS	*mgrB* (3), *phoP* (6), *phoQ* (6), *pmrB* (6)	*mgrB* (3), *phoP* (6), *phoQ* (6), *pmrB* (6)
16	Eltai et al., 2020 [27]	Qatar	2020	Human	13	13	WGS	*mcr-1* (1), *mcr-8* (2), *mgrB* (4), *phoP* (13)	*mgrB* (4), *phoP* (13)
17	Esposito et al., 2018 [28]	Italy	2015–2016	Human	25	25	PCR, sequencing	*crrB* (21), *mgrB* (25), *phoQ* (4), *pmrA* (4), *pmrB* (4)	*crrB* (3), *mgrB* (22), *phoQ* (4), *pmrA* (1), *pmrB* (1)
18	Gentile et al., 2020 [29]	Italy	2013–2014	Human	27	26	NGS, ResFinder	*mgrB* (27), *phoQ* (27), *pmrB* (27)	*mgrB* (14), *phoQ* (12), *pmrB* (2)
19	Giordano et al., 2018 [30]	Italy	2015–2016	Human	29	24	WGS, ResFinder, ISfinder	*mcr-1* (1), *mgrB* (22), *phoP* (2), *pmrA* (3), *pmrB* (3)	*mgrB* (22), *phoP* (2), *pmrA* (3), *pmrB* (3)
20	Haeili et al., 2017 [31]	Iran	2015–2017	Human	20	20	PCR, sequencing	*mgrB* (20), *phoP* (20), *phoQ* (20), *pmrA* (20), *pmrB* (20)	*mgrB* (15), *pmrB* (19)
21	Huang et al., 2021 [32]	Taiwan	2016–2019	Human	24	20	PCR, Sanger sequencing, ISfinder	*crrA* (1), *mgrB* (13), *phoP* (1), *phoQ* (2), *pmrA* (1), *pmrB* (3)	*crrA* (1), *mgrB* (13), *phoP* (1), *phoQ* (2), *pmrA* (1), *pmrB* (3)
22	Jaidane et al., 2018 [33]	Tunisia	2012–2016	Human	13	13	WGS, ResFinder,	*mgrB* (13), *phoP* (13), *phoQ* (13), *pmrA* (13), *pmrB* (13), *pmrC* (13)	*mgrB* (13), *phoQ* (9), *pmrA* (5), *pmrB* (9), *pmrC* (13)
23	Kim & Ko, 2018 [34]	Korea	NA	Human	40	32	PCR, sequencing	*crrA* (4), *crrB* (5), *mgrB* (17), *phoP* (1), *phoQ* (7), *pmrB* (3)	*crrA* (2), *crrB* (5), *mgrB* (17), *phoP* (1), *phoQ* (7), *pmrB* (3)
24	Lagerbäck et al., 2016 [35]	United State	NA	Human	2	2	PCR, sequencing	*mgrB* (1), *pmrB* (2)	*mgrB* (1), *pmrB* (2)
25	Lee et al., 2021 [36]	Korea	2008–2018	Human	2	2	PCR, sequencing	*mgrB* (2), *ompK35* (1), *ompK36* (2), *pmrB* (2), *pmrC* (2), *pmrE* (2), *pmrK* (2)	*mgrB* (2), *ompK35* (1), *ompK36* (2), *pmrB* (2), *pmrC* (2), *pmrE* (2), *pmrK* (2)
26	Leung et al., 2017 [37]	United State	2008–2012	Human	11	9	PCR, NGS	*crrB* (4), *mgrB* (9), *pmrB* (3), *pmrF* (2), *pmrJ* (4), *pmrK* (3)	*crrB* (4), *mgrB* (7), *pmrB* (3), *pmrF* (2), *pmrJ* (1), *pmrK* (1)
27	Liu et al., 2021 [38]	China	2017–2019	Human	53	13	WGS	*mcr-1* (3), *mcr-8* (1), *mgrB* (3), *phoQ* (1), *pmrA* (1), *pmrB* (11)	*mgrB* (3), *phoQ* (1), *pmrA* (1), *pmrB* (11)
28	Longo et al., 2019 [39]	Brazil	2016	Human	23	10	WGS, PROVEAN	*crrB* (3), *mgrB* (10), *phoQ* (10), *pmrB* (10)	*crrB* (3), *mgrB* (7), *phoQ* (6), *pmrB* (9)
29	Lu et al., 2018 [40]	China	2015–2016	Human	5	3	WGS, ResFinder	*mcr-1* (1), *phoQ* (3)	*phoQ* (3)
30	Malli et al., 2018 [41]	Greece	2016–2017	Human	98	75	PCR, sequencing	*mgrB* (98)	*mgrB* (75)
31	Mathur et al., 2018 [42]	India	NA	Human	8	8	WGS	*arnA* (8), *arnB* (4), *arnC* (8), *arnT* (8), *mgrB* (2), *pagP* (6), *phoP* (8), *phoQ* (8), *pmrB* (8), *pmrC* (8), *pmrJ* (6)	*arnA* (8), *arnB* (4), *arnC* (8), *arnT* (8), *mgrB* (2), *pagP* (6), *phoP* (8), *phoQ* (8), *pmrB* (8), *pmrC* (8), *pmrJ* (6)
32	Mirshekar et al., 2020 [43]	Iran	2018–2019	Human	20	4	PCR, sequencing, ISfinder	*mgrB* (20)	*mgrB* (4)
33	Moghimi, Haeili & Mohajjel Shoja, 2021 [44]	Iran	NA	Human	9	5	PCR, sequencing	*mgrB* (9)	*mgrB* (5)
34	Morales-León et al., 2020 [45]	Chile	2011–2014	Human	8	8	WGS, ResFinder, PROVEAN	*mgrB* (4), *phoP* (4), *phoQ* (1), *pmrB* (3)	*mgrB* (4), *phoP* (4), *phoQ* (1), *pmrB* (3)
35	Ngbede et al., 2021 [46]	Nigeria	2016–2019	Human and animal	17	17	WGS, PROVEAN	*arnT* (1), *crrB* (17), *mcr-1* (3), *mcr-8* (5), *mgrB* (17), *ompK36* (10), *ompK37* (17), *ramR* (17)	*arnT* (1), *crrB* (17), *mgrB* (17), *ompK36* (10), *ompK37* (17), *ramR* (17)
36	Otter et al., 2017 [47]	United Kingdom	2014–2015	Human	25	24	WGS	*mgrB* (23), *phoQ* (1)	*mgrB* (23), *phoQ* (1)
37	Palani et al., 2020 [48]	India	2017–2018	Human	25	11	PCR, sequencing	*mgrB* (25)	*mgrB* (11)
38	Poirel et al., 2015 [49]	Multiple countries	NA	Human	47	12	PCR, sequencing, ISfinder	*mgrB* (12)	*mgrB* (12)
39	Pragasam et al., 2017 [50]	India	2013–2015	Human	8	8	PCR, WGS	*arnA* (8), *arnB* (7), *arnC* (8), *arnT* (8), *mgrB* (4), *pagP* (6), *phoP* (8), *phoQ* (8), *phoR* (3), *pmrB* (7), *pmrC* (8)	*arnA* (8), *arnB* (7), *arnC* (8), *arnT* (8), *mgrB* (4), *pagP* (4), *phoP* (8), *phoQ* (8), *phoR* (3), *pmrB* (7), *pmrC* (8)
40	Sato et al., 2020 [51]	Japan	2017	Human	4	3	WGS, ResFinder	*phoP* (1), *pmrB* (2)	*phoP* (1), *pmrB* (2)
41	Seo et al., 2021 [52]	Korea	NA	Human	35	15	Sequencing	*phoP* (14), *phoQ* (10), *pmrB* (9)	*phoP* (14), *phoQ* (10), *pmrB* (9)
42	Shankar et al., 2019 [53]	India	2016–2017	Human	65	19	PCR, sequencing, ISfinder	*mgrB* (12), *phoP* (3) *phoQ* (9)	*mgrB* (12), *phoP* (3), *phoQ* (9)
43	Sharahi et al., 2021 [54]	Iran	2016–2018	Human	16	6	PCR, ISfinder	*mgrB* (16), *phoP* (16), *phoQ* (16), *pmrA* (16), *pmrB* (16)	*mgrB* (6), *phoP* (1), *phoQ* (1), *pmrB* (1)
44	Uz Zaman et al., 2018 [55]	Saudi Arabia	2011–2015	Human	23	23	PCR, Sanger sequencing, ISfinder	*mgrB* (18), *phoP* (6)	*mgrB* (18), *phoP* (6)
45	Venditti et al., 2021 [56]	Italy	2019–2020	Human	6	6	WGS	*mgrB* (6), *ompK35* (6), *ompK36* (6)	*mgrB* (6), *ompK35* (6), *ompK36* (6)
46	Wang et al., 2017 [57]	China	2011–2014	Human and animal	17	16	PCR, WGS	*mcr-1* (4), *mgrB* (17), *phoQ* (17), *pmrB* (17)	*mgrB* (6), *pmrB* (16)
47	Yang et al., 2020 [58]	Taiwan	2012–2015	Human	49	48	PCR, sequencing	*crrB* (28), *mgrB* (32), *phoP* (4), *phoQ* (10), *pmrA* (5), *pmrB* (16)	*crrB* (28), *mgrB* (31), *phoP* (4), *phoQ* (10), *pmrA* (5), *pmrB* (16)
48	Zafer et al., 2019 [59]	Egypt	2016–2017	Human	22	1	PCR, sequencing	*mcr-1* (1), *mgrB* (12)	*mgrB* (1)
49	Zhang et al., 2019 [60]	China	2015	Human	3	3	PCR, sequencing	*pmrB* (3)	*pmrB* (3)
50	Zhu et al., 2019 [61]	Greece	NA	Human	8	8	PCR, Sanger sequencing	*arnB* (1), *mgrB* (8), *phoP* (8), *phoQ* (3), *pmrB* (1), *pmrC* (1),	*arnB* (1), *mgrB* (8), *phoP* (8), *phoQ* (3), *pmrB* (1), *pmrC* (1)

(n): number of isolates. NA: not applicable. NGS: next generation sequencing. PCR: polymerase chain reaction. PROVEAN: Protein Variation Effect Analyzer.

**Table 2:** Subgroup analysis of prevalence of mutated colistin-resistant *K. pneumoniae* (ColRkp) according to countries of studies.

Country of Study	No. of Study	Prevalence (%)	95% CI	*I* ^2^	Q	Heterogeneity Test	
						DF	*p*
Brazil	2	80.8	10.8–99.3	90.805	10.875	1	0.001
Chile	1	94.4	49.5–99.7	0.000	0.000	0	1.000
China	5	71.3	29.3–93.7	79.333	19.355	4	0.001
Croatia	1	42.9	20.6–68.4	0.000	0.000	0	1.000
Egypt	1	4.5	0.6–26.1	0.000	0.000	0	1.000
France	1	63.6	42.3–80.7	0.000	0.000	0	1.000
Greece	3	77.5	69.3–84.1	0.000	1.279	2	0.528
India	5	68.6	37.3–88.9	79.819	19.821	4	0.001
Iran	4	51.0	20.7–80.6	75.566	12.278	3	0.006
Italy	6	88.4	78.6–94.1	28.437	6.987	5	0.222
Japan	1	75.0	23.8–96.6	0.000	0.000	0	1.000
Korea	5	68.6	39.9–87.7	74.004	15.387	4	0.004
Multiple countries	2	41.7	14.9–74.5	91.559	11.847	1	0.001
Nigeria	1	97.2	67.8–99.8	0.000	0.000	0	1.000
Qatar	1	96.4	61.6–99.8	0.000	0.000	0	1.000
Saudi Arabia	1	97.9	74.1–99.9	0.000	0.000	0	1.000
Spain	1	19.0	7.3–41.2	0.000	0.000	0	1.000
Taiwan	3	92.8	73.6–98.3	51.071	4.088	2	0.130
Tunisia	1	96.4	61.6–99.8	0.000	0.000	0	1.000
Turkey	1	72.2	63.3–79.6	0.000	0.000	0	1.000
United Kingdom	1	96.0	76.5–99.4	0.000	0.000	0	1.000
United States	2	82.1	53.9–94.8	0.000	0.004	1	0.952
Vietnam	1	96.8	80.4–99.5	0.000	0.000	0	1.000

## Data Availability

The datasets used and/or analyzed during the current study are included in the manuscript.

## Supplementary Materials

The following supporting information can be downloaded at: https://www.mdpi.com/article/10.3390/tropicalmed7120414/s1, Table S1. Quality of the included studies by the JBI critical appraisal checklist for studies reporting prevalence data. Table S2. Type of mutation in colistin-resistant genes.
