# Supplementary material for: Prevalence of Mutated Colistin-Resistant Klebsiella pneumoniae: A Systematic Review and Meta-Analysis
Source: Trop Med Infect Dis. 2022 Dec 2;7(12):414. doi: 10.3390/tropicalmed7120414 (PMC9782491; doi:10.3390/tropicalmed7120414)
Supplement: Supplementary file 1 [file tropicalmed-07-00414-s001.zip › tropicalmed-1996792-SI.pdf]

Table S1. Quality of the included studies by the JBI critical appraisal checklist for studies reporting prevalence data.

No.	Author	Check List									Score
		1	2	3	4	5	6	7	8	9	
1	Avgoulea *et al.*, 2018 [12]	YES	YES	YES	YES	YES	YES	NO	NO	YES	7
2	Azam *et al.*, 2021 [13]	YES	YES	YES	YES	YES	YES	YES	YES	YES	9
3	Baron *et al.*, 2021 [14]	YES	YES	YES	YES	YES	YES	YES	YES	YES	9
4	Barragán-Prada *et al.*, 2019 [15]	YES	YES	YES	YES	YES	YES	YES	YES	YES	9
5	Berglund *et al.*, 2018 [16]	YES	YES	YES	YES	YES	YES	YES	YES	YES	9
6	Bonura *et al.*, 2015 [17]	YES	YES	YES	YES	YES	YES	YES	YES	YES	9
7	Can *et al.*, 2018 [18]	YES	NO	YES	YES	YES	YES	NO	YES	YES	7
8	Cannatelli *et al.*, 2014 [19]	YES	YES	YES	YES	NO	YES	YES	NO	YES	7
9	Chen *et al.*, 2021 [20]	YES	YES	YES	YES	YES	YES	NO	YES	YES	8
10	Cheng *et al.*, 2016 [21]	YES	YES	YES	YES	YES	YES	YES	YES	YES	9
11	Choi & Ko, 2015 [22]	YES	YES	YES	YES	YES	YES	YES	NO	YES	8
12	Choi & Ko, 2020 [23]	YES	YES	YES	YES	NO	YES	YES	YES	YES	8
13	da Silva *et al.*, 2020 [24]	YES	YES	YES	YES	YES	YES	YES	YES	NO	8
14	Di Tella *et al.*, 2019 [25]	YES	YES	YES	YES	NO	YES	YES	YES	NO	7
15	D’Onofrio *et al.*, 2020 [26]	YES	YES	YES	YES	YES	YES	YES	YES	YES	9
16	Eltai *et al.*, 2020 [27]	YES	YES	YES	YES	NO	YES	NO	YES	YES	7
17	Esposito *et al.*, 2018 [28]	YES	YES	YES	YES	NO	YES	YES	YES	NO	7
18	Gentile *et al.*, 2020 [29]	YES	YES	YES	YES	YES	YES	YES	YES	YES	9
19	Giordano *et al.*, 2018 [30]	YES	YES	YES	YES	YES	YES	YES	NO	YES	8
20	Haeili *et al.*, 2017 [31]	YES	YES	YES	YES	YES	YES	YES	NO	YES	8
21	Huang *et al.*, 2021 [32]	YES	YES	YES	YES	YES	YES	YES	YES	YES	9
22	Jaidane *et al.*, 2018 [33]	YES	YES	YES	NO	NO	YES	YES	YES	YES	7
23	Kim & Ko, 2018 [34]	YES	YES	YES	NO	NO	YES	YES	YES	YES	7
24	Lagerbäck *et al.*, 2016 [35]	YES	YES	YES	YES	YES	YES	YES	YES	YES	9
25	Lee *et al.*, 2021 [36]	YES	YES	YES	YES	NO	YES	YES	YES	NO	7

26	Leung *et al.*, 2017 [37]	YES	YES	YES	YES	YES	YES	YES	NO	YES	8
27	Liu *et al.*, 2021 [38]	YES	YES	YES	YES	NO	YES	YES	YES	NO	7
28	Longo *et al.*, 2019 [39]	YES	YES	YES	YES	YES	YES	YES	YES	YES	9
29	Lu *et al.*, 2018 [40]	YES	YES	YES	YES	YES	YES	NO	YES	YES	8
30	Malli *et al.*, 2018 [41]	YES	YES	YES	YES	YES	NO	YES	YES	YES	8
31	Mathur *et al.*, 2018 [42]	YES	YES	YES	YES	NO	YES	YES	YES	NO	7
32	Mirshekar *et al.*, 2020 [43]	YES	YES	YES	YES	YES	YES	YES	YES	YES	9
33	Moghimi, Haeili & Mohajjel Shoja, 2021 [44]	YES	YES	YES	NO	YES	YES	YES	YES	YES	8
34	Morales-León *et al.*, 2020 [45]	YES	YES	YES	YES	YES	YES	YES	YES	YES	9
35	Ngbede *et al.*, 2021 [46]	YES	YES	YES	YES	YES	YES	YES	YES	YES	9
36	Otter *et al.*, 2017 [47]	YES	YES	YES	YES	YES	YES	YES	YES	YES	9
37	Palani *et al.*, 2020 [48]	YES	YES	YES	YES	YES	YES	YES	YES	YES	9
38	Poirel *et al.*, 2015 [49]	YES	YES	YES	NO	NO	YES	YES	YES	YES	7
39	Pragasam *et al.*, 2017 [50]	YES	YES	YES	YES	NO	YES	YES	YES	NO	7
40	Sato *et al.*, 2020 [51]	YES	YES	YES	YES	YES	YES	NO	YES	NO	
41	Seo *et al.*, 2021 [52]	YES	YES	YES	NO	YES	YES	YES	YES	YES	8
42	Shankar *et al.*, 2019 [53]	YES	YES	YES	YES	NO	YES	YES	YES	NO	7
43	Sharahi *et al.*, 2021 [54]	YES	YES	YES	YES	NO	YES	YES	YES	NO	7
44	Uz Zaman *et al.*, 2018 [55]	YES	YES	YES	YES	YES	YES	YES	YES	YES	9
45	Venditti *et al.*, 2021 [56]	YES	YES	YES	YES	YES	YES	YES	NO	YES	8
46	Wang *et al.*, 2017 [57]	YES	YES	YES	YES	NO	YES	YES	YES	NO	7
47	Yang *et al.*, 2020 [58]	YES	YES	YES	YES	YES	YES	YES	YES	YES	9
48	Zafer *et al.*, 2019 [59]	YES	YES	YES	YES	YES	YES	NO	YES	YES	8
49	Zhang *et al.*, 2019 [60]	YES	YES	YES	YES	YES	YES	YES	YES	YES	9
50	Zhu *et al.*, 2019 [61]	YES	YES	YES	YES	NO	YES	YES	YES	NO	7

- Checklist questions: 1). Was the sample frame appropriate to address the target population?; 2). Were study participants sampled in appropriate way?; 3). Was the sample size adequate?; 4). Were the study subjects and the setting described in?; 5). Was a sample size justification, power description, or variance and effect estimates provided?; 6). Were valid methods used for the identification of the condition?; 7). Was the condition measured in a standard, reliable way for all participants?; 8). Was there appropriate statistical analysis?; 9). Was the response rate adequate, and if not, was the low response rate managed appropriately?
- Score: '1' for 'yes', '0' for 'no'; score '7' to '9' were of sufficient quality.

Table S2. Type of mutation in colistin resistant genes.

No.	Study ID (ref)	Gene (n)	Mutation/Deletion/Insertion (n)
1	Avgoulea *et al.*, 2018 [12]	*mgrB* (15)	Insertional inactivation of ISKpn26 at nt 85 (15)
2	Azam *et al.*, 2021 [13]	*mgrB* (4)	82T>G (C28G) (1); ins. Of: IS1 at 116-117 (2) and IS5 at 94-95 (1)
		phoP (1)	Thr151Ala (1)
		phoQ (4)	Leu30Gln (1), Ala351Asp (1), deletion (87-90) (1), deletion (267-268) (1)
		pmrA (1)	Gly53Ser (1)
		pmrB (7)	Gly250Cys (3), Ala252Gly (3), Asp150Val (1), Leu237Arg (1), His267Pro (2), Arg315Pro (1), Gln331His (1), Thr157Pro (1)
3	Baron *et al.*, 2021 [14]	*acrS* (12)	Ser76Arg (9), His79Gln (2), Pro211Ser (1)
		crrB (10)	Cys68Ser (10)
		mgrB (2)	Stop codon at the codon for the 13th amino acid (1), IS10 at nt 76 (1)
		phoP (1)	Leu12Gln (1)
		phoQ (2)	Leu87Pro (1), Asn135Lys (1)
		pmrA (13)	Ala41Thr (9), Gly53Val (1), Ala217Val (2), Ser64Thr (1)
		pmrB (11)	Leu213Met (9), Thr246Ala (11)
4	Barragán-Prada *et al.*, 2019 [15]	*mgrB* (3)	Ins. of ISKpn18 (1) and ISKpn14 (2)
		pmrA (1)	Gly53Ser (1)
		pmrB (1)	Thr140Pro (1)
5	Berglund *et al.*, 2018 [16]	*mgrB* (30)	58T>G (W20G) (8); non-stop substitution 58T>C (3); frameshift deletion (ΔC70) (7); insertional inactivation of: IS10R between nt-26 and -27 (2), ISKpn26 between nt 74 and 75 (3), IS10R between nt 76 and 77 (1), IS903 between nt 93 and 94 (6)
6	Bonura *et al.*, 2015 [17]	*mgrB* (32)	11T>A (non-sense, premature termination) (12); 139T>C (Trp47Arg) (2); 59G>T (Trp20Leu) (1); 64C>T (non-sense, premature termination) (1); insertional inactivation of: IS5 at nt 75 (3), IS1F at nt 105 (2) and at nt 61 (11)
7	Can *et al.*, 2018 [18]	*mgrB* (83)	Point mutations (83), deletions (6), ins. of IS (IS903, IS5, IS1) (77)

8	Cannatelli *et al.*, 2014 [19]	*mgrB* (39)	Insertional inactivation of: IS5 at nt 75 (RW) (7), at nt 126 (FW) (1), at nt 75 (RW) (10), IS1F at nt 105 (FW) (2), ISKpn14 at nt 124 (FW) (2); 71T>A (Leu24His) (1); 109G>A (Gly37Ser) (5); 83G>A (Cys28Tyr) (1); Δ18/27 (frameshift and premature termination) (2); 7A>T (nonsense, premature termination) (1); Δg47 (frameshift and premature termination) (2); Δ109/119 (frameshift and premature termination) (1); ΔmgrB (from - 400 to + 599) (1); ΔmgrB locus (3)
9	Chen *et al.*, 2021 [20]	*mgrB* (2)	Insertional inactivation of: IS1 between nt −29 and −30 (1), ISKpn14 between nt −29 and −30 (1)
10	Cheng *et al.*, 2016 [21]	*crrB* (8)	Trp140Arg (1), Asn141Ile (2), Pro151Ser (1), Ser195Asn (1), Gln10Leu (2), Tyr31His (1)
11	Choi & Ko, 2015 [22]	*phoP* (4)	Val26Leu (4)
		phoQ (12)	Asn253Asp (1), Tyr268Ser (4), Tyr268Cys (4), Asp418Asn (4), deletion at nt 341-352 (4)
		pmrB (12)	Phe344Leu (4), ins. 46-bp at nt 256-302 (8)
12	Choi & Ko, 2020 [23]	*mgrB* (2)	Ins. of IS5 (2)
		phoQ (1)	803A>C (Tyr268Ser) (1), 12-bp deletion at nt 341 (1)
13	da Silva *et al.*, 2020 [24]	*mgrB* (29)	Repeated sequence at nt 89 (6); insertional inactivation of: ISEcp1 at nt 124 (3), ISKpn13 at nt 125 (2), nt 75 (1) and nt 89 (1), ISKpn18 at nt 122 (1), IS5-like element at nt 89 (6) and nt 75 (1), IS903 at nt 89 (6) and nt 88 (1); 7A>T (1)
14	Di Tella *et al.*, 2019 [25]	*mgrB* (18)	62C>A (Thr21Asn) (1); 95T>G (Val32Gly) (8); 60G>A (Trp20Stop) (1); insertional inactivation of: IS5 (5) and ISKpn14 (1); Δg19 (deletion of guanine at position 19) (2)
13	D'Onofrio *et al.*, 2020 [26]	*mgrB* (3)	Lys3X (2), disrupted by IS26 transposon at G7 (1)
		phoP (6)	Leu26Gln (6)
		phoQ (6)	Asp150Gly (6), Leu482Gln (2), Ser475Gly (2)
		pmrB (6)	Arg256Gly (5), Pro167Thr (1), Val177Arg (2), Gln356Ala (1), Ala246Thr (1)
16	Eltai *et al.*, 2020 [27]	*mgrB* (4)	Interruption ISKpn26 (IS5) at nt 74 (1), ins. at nt 74 (cause frameshift) (1), interruption at nt 118 (1), absence of mgrB (1)
		phoP (13)	Leu26Gln (13)
17	Esposito *et al.*, 2018 [28]	*crrB* (3)	Leu296Gln (3), Gln287Lys (1)
		mgrB (22)	95T>G (Val32Gly) (1); 50T>G (Leu17Arg) (1); 139T>A (Trp47Arg) (1); 88C>T (non-sense, premature termination) (4); insertional inactivation of: IS5 at nt 75 (4), IS903 at nt 69 (1); Δg19 (frameshift mutation) (10)
		phoQ (4)	Asp150Gly (4), Trp215Gly (1), Leu257Pro (1)
		pmrA (1)	Ala217Val (1)

		pmrB (1)	Arg256Gly (1)
18	Gentile *et al.*, 2020 [29]	*mgrB* (14)	88C>T (1), Δnt61/70 (10-nt deletion) (8), ins. of IS5 between nt 74 and 75 (1), ΔmgrB promoter (1), -55ΔG (1)
		phoQ (12)	168C>A (Ser56Arg) (1), 260T>C (Leu87Pro) (1), ins. 799/801 (3-nt insertion (GAC)) (10)
		pmrB (2)	137T>A (Val46Glu) (1), 284C>T (Pro95Leu) (1)
19	Giordano *et al.*, 2018 [30]	*mgrB* (22)	Δ109–119 (16), ISKpn26 at nt 75 (2), ISKpn25 at nt 133 (2), 7A>Tstop codon (2)
		phoP (2)	602T>C (Ile201Thr) (1), 55C>A (1)
		pmrA (3)	121G>A (Ala41Thr) (2), 121C>T (1)
		pmrB (3)	385G>C (Ala129Pro) (1), 637A>C (1), 766G>C (2), 245G>T (2)
20	Haeili *et al.*, 2017 [31]	*mgrB* (15)	Insertional inactivation of: IS1-like at nt +120 (3) and at promoter region (nt -60) (1), IS5-like at nt +70 (2); premature termination by nonsense mutation at nt 88 (7) and at nt 117 (2)
		pmrB (19)	Ala246Thr (7), Leu213Met (7), Arg256Gly (5)
21	Huang *et al.*, 2021 [32]	*crrA* (1)	Ins. IS5 at nt 65 (1)
		mgrB (13)	Thr21Pro (1); ins. of: IS903B at nt 95 (1), IS26 at nt 28 (1), ISKpn14 at nt 57 (1) and nt 120 (1), ISKpn26 at nt 80 (1), nt 126 (1) and nt 75 (1), IS1R at nt 117 (1) and nt 78 (1), IS10R at nt 77 (1); ΔC48 (deletion of cytidine at nt 48-cause frameshift) (1); Gln30stop (1)
		phoP (1)	Val122Phe (1)
		phoQ (2)	Leu96Pro (1), Ala214Ser (1)
		pmrA (1)	Gly53Cys (1)
		pmrB (3)	Thr157Pro (2), Leu245Arg (1)
22	Jaidane *et al.*, 2018 [33]	*mgrB* (13)	Insertional inactivation IS1 (2), ΔmgrB (1), ΔK2-V7 (1), ΔT41 (1), ΔT40 (1), ΔI41 (1), ΔI40 (1), Δ24N (1), Phe28Cys (10)
		phoQ (9)	ΔK2-L6 (9)
		pmrA (5)	Ala217Val (5)
		pmrB (9)	Thr246Ala (9), Arg256Gly (4), Thr157Pro (1)
		pmrC (13)	Val138Ile (13), Ala148Thr (13), Ser204Phe (13), Leu257Ser (4), Glu354Lys (13), Gly469Val (13), Ser25Gly (6), Phe27Cys (3), Leu50Val (2), Pro135Ala (2), Arg319Gln (7)
23	Kim & Ko, 2018 [34]	*crrA* (2)	Asp187Asn (2), Lys217Arg (2)
		crrB (5)	Ile66Val (2), Gln239His (2), Ser93Gly (2), Leu133del (1)
		mgrB (17)	Asn25Ile (1), Trp47Leu (4), ins. of: ISKpn14 at nt -29 (2), nt 142 (1), nt 73 (1) and nt 126 (1), ISkpn26 at nt 32 (1) and nt 75 (1), IS102 at nt 70 (3), IS5 at nt 75 (1); non-stop codon mutation (3); Gly37Val (1)

		phoP (1)	Trp84Cys (1)
		phoQ (7)	Leu173Pro (1), Ser260Asn (1), Asp148Asn (1), Thr244Asn (3), Ser260Ile (1)
		pmrB (3)	Thr157Pro (3)
24	Lagerbäck *et al.*, 2016 [35]	*mgrB* (1)	116G>A (Cys39Tyr) (1)
		pmrB (2)	736G>A (Ala246Thr) (2), 766G>C (Gly256Arg) (2)
25	Lee *et al.*, 2021 [36]	*mgrB* (2)	Ins. of ISs at nt 51 (1) and nt 117 (1)
		ompK35 (1)	1-bp deletion at position 54 (1)
		ompK36 (2)	1-bp insertion at position 419 (1), nonsense mutation after the start codon (1)
		pmrB (2)	Thr246Ala (2), Arg256Gly (2)
		pmrC (2)	Cys27Phe (2), Ile138Val (2), Gln319Arg (2)
		pmrE (2)	Val17Ile (1), Ala33Ser (1), His68Gln (1), Thr105Ala (1), Ala165Glu (1), Asp172Asn (1), Ala274Gly (1), Asn354Asp (1), Glu371Asp (1), Thr373Ala (2), Asn354Ala (1)
		pmrK (2)	Met114Leu (2), Val117Ile (2), Arg372Lys (2), Gly462Arg (1)
26	Leung *et al.*, 2017 [37]	*crrB* (4)	Leu94Met (1), Pro151Gln (1), Leu296Gln (1), Leu133Arg (1)
		mgrB (7)	Gln30Arg (1); ins. ISKpn26-like at nt 75 (2) and IS903B-like at nt 70 (1); nt19frameshift (1); Gln30stop (1)
		pmrB (3)	His340Arg (1), Ser85Arg (1), Thr157Pro (1)
		pmrF (2)	Phe280Leu (1), Lys322Gln (1)
		pmrJ (1)	Glu25Ala (1), Arg29Lys (1), Ile53Val (1), Leu94Ile (1)
		pmrK (1)	Ile117Val (1), His156Gln (1), Asp441Glu (1)
27	Liu *et al.*, 2021 [38]	*mgrB* (3)	ΔmgrB (2), Δpromoter area (1)
		phoQ (1)	Val24Gly (1)
		pmrA (1)	Met66Ile (1), Glu35Ala (1)
		pmrB (11)	Thr246Ala (11), Arg256Gly (3), Ser203Pro (1), Thr157Pro (1), Val257Ala (1), Thr240Met (1), Met285Leu (1)
28	Longo *et al.*, 2019 [39]	*crrB* (3)	Cys68Ser (3), Ser195Asn (1), Gln296Leu (3)
		mgrB (7)	Insertional inactivation of: ISKPn25 (1), IS903 (1) and IS5 (2); deletion of nt 17 and 18 (3)
		phoQ (6)	Asp90Glu (2), Thr84Lys (2), Leu37Pro (1), His410Tyr (3), Leu26del (2), Val27del (2)
		pmrB (9)	Arg256Gly (5), Thr246Ala (8), Pro95Leu (1)
29	Lu *et al.*, 2018 [40]	*phoQ* (3)	Asp150Gly (3)

30	Malli *et al.*, 2018 [41]	*mgrB* (75)	83G>A (Cys28Tyr) (1); 3G>A (Met1Val) (4); 103T>A (Phe35Ile) (1); insertional inactivation of: IS903B at nt +70 (FW) (3), ISEc68 at nt +75 (FW) (2), ISEcp1-blaCTXM15 at nt +125 (FW) (1) and at nt +21 (RW) (1), ISKpn14 at nt +72 (RW) (2) and at nt +43 (FW) (8), IS5 at nt +33 (RW) (2), IS5 at +75 (RW) (15); premature stop codon at 3rd a.a. (20) and 8th a.a. (1); expression interfering: ISKpn26 at nt -39 (1) and ISKpn14 at nt -11 (1); partial or complete deletion of mgrB locus (11), 142A>C (Stop48Glu) (1)
31	Mathur *et al.*, 2018 [42]	*arnA* (8)	Ile260Leu (8), Asn442Lys (8)
		arnB (4)	Gly47Asp (2), Ala112Asp (4), Ile126Val (2), Asp285Glu (2)
		arnC (8)	Ser30Thr (6), Ser19Thr (2)
		arnT (8)	Gln156His (8), Arg157Ser (7), Arg372Lys (6), Arg158Ser (1), Ile474Asn (2)
		mgrB (2)	Val1Ala (1), Leu24His (1)
		pagP (6)	Phe170Ile (4), Phe190Ile (1), Phe189Ile (1)
		phoP (8)	Arg114Ala (8)
		phoQ (8)	Asp150Gly (8)
		pmrB (8)	Asp150His (4), Arg256Gly (4), Leu344Pro (8), Thr157Pro (2), Ala246Thr (2)
		pmrC (8)	Cys27Phe (4), Val39Leu (7), Ala279Gly (2), Gln319Arg (4), Val42Leu (1), Ser260Leu (1), Ser257Leu (1), Arg152His (2), Asp477Asn (2)
		pmrJ (6)	Trp52Leu (1), Val53Ile (1), Ile94Leu (4), Ile300Val (4), Ser164Pro (2)
32	Mirshekar *et al.*, 2020 [43]	*mgrB* (4)	Insertional inactivation of IS903B (1), premature stop codon at 21st amino acid (3)
33	Moghimi, Haeili & Mohajjel Shoja, 2021 [44]	*mgrB* (5)	Ins. of IS5 between +51 and +52 (1), IS3 between +112 and +113 (1); ΔT72 (frameshift) (1); nonsense mutation at nt88 (2)
34	Morales-León *et al.*, 2020 [45]	*mgrB* (4)	Ins. of IS5 (1) and IS1 (2); cys39stop (1)
		phoP (4)	Thr104Ala (4)
		phoQ (1)	Ala351Asp (1)
		pmrB (3)	Pro95Leu (1), Arg256Gly (2)
35	Ngbede *et al.*, 2021 [46]	*arnT (1)*	Gly164Ser (1)
		crrB (17)	Trp140Ser (1), Asn141His (2), Asn195Ser (14), Trp140Leu (2), Asn141Ile (1), Leu56del (1), Ala52del (1), Ala59del (1)

		mgrB (17)	Met1Val (17)
		ompK36 (10)	Ala217Ser (9), Asn218His (4)
		ompK37 (17)	Ile70Met (17), Ile128Met (17), Asn230Gly (11), Thr261Ala (1), ins. of 4 amino acids between 233 and 234 (11)
		ramR (17)	Met1Val (17)
36	Otter *et al.*, 2017 [47]	*mgrB* (23)	Cys16Stop (16), Lys3Stop (7)
		phoQ (1)	Leu396Gln (1)
37	Palani *et al.*, 2020 [48]	*mgrB* (11)	88C>T (premature termination) (1), ΔmgrB (8), ins. of ISKpn14 with complete deletion (1), insertional inactivation of ISKpn26 between +30 and +31 (1)
38	Poirel *et al.*, 2015 [49]	*mgrB* (12)	Insertional inactivation of: IS5 between nt +74 and +75 (5), ISKpn13 between nt +74, +75 (1), ISKpn14 between nt +127, +128 (1), IS10R between nt -27 and -26 (2); premature stop codon (3)
39	Pragasam *et al.*, 2017 [50]	*arnA* (8)	Ile260Leu (8), Asn442Lys (8), Leu161Cys (1), Ser18Ala (1), Thr185Ala (1)
		arnB (7)	Gly47Asp (4), Ala112Asp (7), Ile126Val (4), Asp285Glu (3)
		arnC (8)	Ser19Thr (3), Ser30Thr (5)
		arnT (8)	Ala55Gly (8), Ser56Leu (8), Ala57Arg (8), Thr58Tyr (8), TYr59Phe (8), Lys372Arg (3), Ile474Asn (3), Leu114Met (1), Ile117Val (1), His156Gln (1)
		mgrB (4)	truncated protein 27 amino acid (2), Δa10 (deletion of A at position 10) (2)
		pagP (4)	Phe170Ile (3), Phe170Thr (1)
		phoP (8)	Arg114Ala (7), Arg128Ala (1)
		phoQ (8)	Asp150Gly (3), Asp146Gly (5)
		phoR (3)	Arg69Cys (3)
		pmrB (7)	Ala246Thr (3), Leu344Pro (7), Thr157Pro (1), Arg256Gly (1)
		pmrC (8)	Val39Leu (5), Ser257Leu (2), Ala279Gly (5), Val42Leu (3), Ser260Leu (2), Arg152His (2), Asp477Asn (2), Arg155His (1), Asp480Asn (1), Cys27Phe (1), Val50Leu (1), Ala135Pro (1)
40	Sato *et al.*, 2020 [51]	*phoP* (1)	Glu82Leu (1)
		pmrB (2)	Ser203Pro (2)
41		*phoP* (14)	Arg171Lys (14), Met175Leu (1), Gln177Arg (1)

	Seo *et al.*, 2021 [52]	*phoQ* (10)	Arg359Lys (7), Asp152His (1), Ser168Asn (1), Ala200Gly (1), Asn202His (1), Leu62Pro (1), Lys46Asn (1), Lys359Arg (1), Cys395Ser (1), Thr407Ser (1)
		pmrB (9)	Thr157Pro (1), Lys220Glu (1), Glu220Lys (7), His221Pro (7), Thr157Pro (2), Gly161Glu (1)
42	Shankar *et al.*, 2019 [53]	*mgrB* (12)	Met1Arg (1); Cys28Gly (2); elongated mgrB of 55 a.a. (1); truncated by: IS1R (1), IS903B (1), ISKpn14 (1), ISKpn26 (1) and IS102 (1); premature stop codon at 21 a.a. and change in the sequence of protein after 10th amino acid (3)
		phoP (3)	Ala114Arg (1), Thr151Ala (1), Glu22Lys (1)
		phoQ (9)	Leu209Cys (1), Trp161Leu (2), Gly117Asp (1), Val370Glu (1), Leu172Gln (1), Trp182Ser (1), Phe445Gly (1), Pro424Leu (1), Val446Trp (1), Δval444 (1), changes from 218 to 239 and 436 to 448 a.a. (1)
43	Sharahi *et al.*, 2021 [54]	*mgrB* (6)	88C>T (non-sense, premature termination) (4), 117C>A (non-sense, premature termination) (1), insertional inactivation of IS5 at nt 75 (1)
		phoP (1)	171A>C (Glu57Asp) (1)
		phoQ (1)	449 A>G (Asp150Gly) (1)
		pmrB (1)	469A>C (Thr157Pro) (1)
44	Uz Zaman *et al.*, 2018 [55]	*mgrB* (18)	ΔT22 (Deletion 'T' at position +22) (1); ins. ISKpn14 (10), IS903 (3) and ISKpn28 (4)
		phoP (6)	Leu26Gln (6)
45	Venditti *et al.*, 2021 [56]	*mgrB* (6)	Ins. of ISKpn26 (5), stop codon at a.a. position 4 (1)
		ompK35 (6)	Ins. of guanine in nt positions 121 and 399 (6)
		ompK36 (6)	Ins. mutation of glycine-aspartic acid residues (GD) at a.a. positions 134-135 in the ompK36 L3 loop (6)
46	Wang *et al.*, 2017 [57]	*mgrB* (6)	Gln22Pro (6)
		pmrB (16)	Ala5Val (3), Asn105Ser (3), Met175Val (3), Ala228Thr (3), Arg256Gly (13)
47	Yang *et al.*, 2020 [58]	*crrB* (28)	Asn141Tyr (1), Leu94Met (1), Asp57Val (1), Gln10Leu (1), Gln287Lys (3), Gln99Arg (3), Gln239His (3), Leu295Phe (3), Asp225Glu (1), deletion (19)
		mgrB (31)	Insertional inactivation of: IS10R at nt76 (5), ISKpn26 at nt74 (8), ISKpn14 at nt119 (1), ISEcpl at nt127 (1), IS903B at nt44 (1), 4 base fragment (5'-TGT-3') at nt58 (1); Δnt1-22 (truncated) (1); Δnt22-32 (frameshift) (1); premature termination by nonsense mutation at nt30 (2), nt90 (2), nt 12 (1) and nt 24 (1); deletion (6)
		phoP (4)	Arg48Lys (3), Glu96Lys (1)
		phoQ (10)	Arg64Lys (2), Gln92Lys (2), Ala106Thr (2), Glu112Asp (2), Ile139Val (2), Leu163Phe (2), Val196Ile (3), Thr372Ser (3), Ala22Thr (4), Ile422Leu (5), Ala63Glu (1), His410Tyr (1), Δnt326-349 (1)

		pmrA (5)	Thr32Asn (1), Gln73His (1), Ala41Thr (3), Gly53Val (1)
		pmrB (16)	Thr140Pro (1), Val89Gly (1), Gln100Arg (1), Asn105Ser (3), Ala228Thr (3), Gln232Glu (2), Ile242Val (3), Asn244Ser (3), Glu272Gln (3), Leu213Met (3), Ala66Lys (1), Asp150Val (3), Asp178Asn (1), Lys39Arg (1), Ala246Thr (2), Thr157Pro (1)
48	Zafer *et al.*, 2019 [59]	*mgrB* (1)	(GCC>GAC) Pro178Tyr (1); silent mutation (TAA>CAA) at position 144 (1) and TCC>CCC at position 156 (1)
49	Zhang *et al.*, 2019 [60]	*pmrB* (3)	Leu82Arg (3), Arg256Gly (3), Leu344Pro (1)
50	Zhu *et al.*, 2019 [61]	*arnB* (1)	-58A>G (1)
		mgrB (8)	Ins. of ISKpn26 at nt 75 (8)
		phoP (8)	158T>G (Val53Gly) (7), -38G>T (1)
		phoQ (3)	758A>C (Asn253Thr) (2), 1312G>C (Asp438His) (1), 1315A>C (Thr439Pro) (1), Asn253Pro (1)
		pmrB (1)	418A>C (Thr140Pro) (1)
		pmrC (1)	919G>T (Glu307Stop) (1)

(n): number of isolates, a.a.: amino acid, Ins.: insertion, IS: insertion sequences, nt: nucleotide, Δ: deletion.
